# Supplementary material for: 30-day unplanned readmission rate in otolaryngology patients: A population-based study in Thuringia, Germany
Source: PLoS One. 2019 Oct 17;14(10):e0224146. doi: 10.1371/journal.pone.0224146 (PMC6797198; doi:10.1371/journal.pone.0224146)
Supplement: S1 Table — (DOCX) [file pone.0224146.s001.docx]

**S1 Table**

| **S1 Table. Overview about all patients: 15,271 inpatient cases of 12,859 patients in 2015.** | | | |
| --- | --- | --- | --- |
| **Parameter** | **N** | **%** | |
| **Gender** |  |  | |
| Female | 6215 | 40.7 | |
| Male | 9065 | 59.3 | |
| **Localization of the primary disease** |  |  | |
| Pharynx/ cavity of the mouth | 5125 | 31.2 | |
| Ear | 3174 | 19.3 | |
| Paranasal sinus | 2124 | 12.9 | |
| Larynx | 1125 | 6.8 | |
| Outside head and neck region | 1024 | 6.2 | |
| Face/ skin | 957 | 5.8 | |
| Nose | 732 | 4.5 | |
| Neck | 677 | 4.1 | |
| Salivary gland | 575 | 3.5 | |
| Trachea/ lung | 431 | 2.6 | |
| Esophagus | 291 | 1.8 | |
| Head/neck, not otherwise specified | 82 | 0.5 | |
| Eye | 70 | 0.4 | |
| Thyroid | 57 | 0.3 | |
| **ICD-code** |  |  | |
| Respiratory system diseases, ICD: J00-J99 | 6087 | 37.0 | |
| Eye/ ear diseases, ICD: H00-H95 | 2898 | 17.6 | |
| Malignant diseases, ICD: C00-C97 | 2485 | 15.1 | |
| Injury, poisoning and certain other consequences of external causes, ICD: S00-T98 | 944 | 5.7 | |
| Symptoms, signs, abnormal findings, ill-defined causes, not otherwise classified, ICD: R00-R99 | 902 | 5.5 | |
| Benign, in-situ, uncertain neoplasm, ICD: D00-D48 | 730 | 4.4 | |
| Gastrointestinal tract diseases, ICD: K00-K93 | 599 | 3.6 | |
| Nervous system diseases, ICD: G00-G99 | 512 | 3.1 | |
| Certain infectious and parasitic diseases, ICD: A00-B99 | 262 | 1.6 | |
| Musculoskeletal system/connective tissue diseases, ICD: M00-M99 | 214 | 1.3 | |
| Skin and subcutaneous tissue diseases, ICD: L00-L99 | 172 | 1.0 | |
| Circulatory system diseases, ICD: I00-I99 | 150 | 0.9 | |
| Congenital malformations and chromosomal abnormalities, ICD: Q00-Q99 | 145 | 0.9 | |
| Blood forming organ diseases, ICD: D50-D90 | 115 | 0.7 | |
| Factors influencing good health and other utilization of the health care system, ICD: Z00-Z99 | 110 | 0.7 | |
| Endocrine and metabolic diseases, ICD: E00-E90 | 58 | 0.4 | |
| Mental and behavioral disorder, ICD: F00-F99 | 58 | 0.4 | |
| Genitourinary system diseases, ICD: N00-N99 | 3 | <0.1 | |
| **PCCL** |  | |  |
| 0 | 9793 | | 64.1 |
| 1 | 1407 | | 9.2 |
| 2 | 655 | | 4.3 |
| 3 | 668 | | 4.4 |
| 4 | 396 | | 2.6 |
| Missing | 2354 | | 15.4 |
| **DRG Partition** |  |  | |
| Surgical | 9554 | 62.6 | |
| Medical | 5632 | 36.9 | |
| Other | 85 | 0.6 | |
|  | **Mean±SD** | **Median** | |
| Age, years | 47.99±24.22 | 53 | |
| Secondary diagnoses, n | 3.84±3.93 | 3 | |

ICD = International Classification of Diseases; PCCL = Patient Clinical Complexity
