# Supplementary material for: 30-day unplanned readmission rate in otolaryngology patients: A population-based study in Thuringia, Germany
Source: PLoS One. 2019 Oct 17;14(10):e0224146. doi: 10.1371/journal.pone.0224146 (PMC6797198; doi:10.1371/journal.pone.0224146)
Supplement: S2 Table — (DOCX) [file pone.0224146.s002.docx]

**S2 Table**

| **S2 Table. Comparison of the group of patients with 30-day readmission to the group of patients without readmission.** | | | | | | |
| --- | --- | --- | --- | --- | --- | --- |
| **Parameter** | **All** | **Readmission** | | **No readmission** | | **p** |
|  | **N** | **N** | **%** | **N** | **%** |  |
| **All** | 15271 | 1173 | 7.7 | 14098 | 92.3 |  |
| **Gender** |  |  |  |  |  | <0.001 |
| Male | 9056 | 869 | 9.6 | 8187 | 90.4 |  |
| Female | 6215 | 304 | 4.9 | 5911 | 95.1 |  |
| **Localization of the primary disease** | | | | | | |
| Eye |  |  |  |  |  | 0.536 |
| Yes | 70 | 4 | 5.7 | 66 | 94.3 |  |
| No | 15201 | 1169 | 7.7 | 14032 | 92.3 |  |
| Ear |  |  |  |  |  | <0.001 |
| Yes | 3053 | 118 | 3.9 | 2935 | 96.1 |  |
| No | 12218 | 1055 | 8.6 | 11163 | 91.4 |  |
| Nose |  |  |  |  |  | 0.573 |
| Yes | 688 | 49 | 7.1 | 639 | 92.9 |  |
| No | 14583 | 1124 | 7.7 | 13459 | 92.3 |  |
| Paranasal sinus |  |  |  |  |  | <0.001 |
| Yes | 2104 | 54 | 2.6 | 2050 | 97.4 |  |
| No | 13167 | 1119 | 8.5 | 12048 | 91.5 |  |
| Pharynx/ Cavity of mouth |  |  |  |  |  | <0.001 |
| Yes | 4657 | 526 | 11.3 | 4131 | 88.7 |  |
| No | 10614 | 647 | 6.1 | 9967 | 93.9 |  |
| Larynx |  |  |  |  |  | <0.001 |
| Yes | 963 | 160 | 16.6 | 803 | 83.4 |  |
| No | 14308 | 1013 | 7.1 | 13295 | 92.9 |  |
| Trachea/ Lung |  |  |  |  |  | 0.251 |
| Yes | 391 | 36 | 9.2 | 355 | 90.8 |  |
| No | 14880 | 1137 | 7.6 | 13743 | 92.4 |  |
| Salivary gland |  |  |  |  |  | 0.109 |
| Yes | 544 | 32 | 5.9 | 512 | 94.1 |  |
| No | 14727 | 1141 | 7.7 | 13586 | 92.3 |  |
| Neck |  |  |  |  |  | <0.001 |
| Yes | 608 | 71 | 11.7 | 537 | 88.3 |  |
| No | 14663 | 1102 | 7.5 | 13561 | 92.5 |  |
| Face/ Skin |  |  |  |  |  | 0.008 |
| Yes | 908 | 49 | 5.4 | 859 | 94.6 |  |
| No | 14363 | 1124 | 7.8 | 13239 | 92.2 |  |
| Esophagus |  |  |  |  |  | 0.623 |
| Yes | 284 | 24 | 8.5 | 260 | 91.5 |  |
| No | 14987 | 1149 | 7.7 | 13838 | 92.3 |  |
| Thyroid |  |  |  |  |  | 0.557 |
| Yes | 54 | 3 | 5.6 | 51 | 94.4 |  |
| No | 15217 | 1170 | 7.7 | 14047 | 92.3 |  |
| Head/ neck, not otherwise specified |  |  |  |  |  | 0.745 |
| Yes | 81 | 7 | 8.6 | 74 | 91.4 |  |
| No | 15190 | 1166 | 7.7 | 14024 | 92.3 |  |
| Other localization |  |  |  |  |  | <0.001 |
| Yes | 866 | 40 | 4.6 | 826 | 95.4 |  |
| No | 14405 | 1133 | 7.9 | 13272 | 92.1 |  |
| **ICD-code** |  |  |  |  |  |  |
| Certain infectious and parasitic diseases, ICD: A00-B99 |  |  |  |  |  | 0.002 |
| Yes | 251 | 6 | 2.4 | 245 | 97.6 |  |
| No | 15020 | 1167 | 7.8 | 13853 | 92.2 |  |
| Malignant diseases, ICD: C00-C97 |  |  |  |  |  | <0.001 |
| Yes | 1801 | 665 | 36.9 | 1136 | 63.1 |  |
| No | 13470 | 508 | 3.8 | 12962 | 96.2 |  |
| Benign, in-situ, uncertain neoplasm, ICD: D00-D48 |  |  |  |  |  | 0.378 |
| Yes | 702 | 60 | 8.5 | 642 | 91.5 |  |
| No | 14569 | 1113 | 7.6 | 13456 | 92.4 |  |
| Blood forming organ diseases, ICD: D50-D90 |  |  |  |  |  | 0.266 |
| Yes | 104 | 11 | 10.6 | 93 | 89.4 |  |
| No | 15167 | 1162 | 7.7 | 14005 | 92.3 |  |
| Endocrine and metabolic diseases, ICD: E00-E90 |  |  |  |  |  | 0.492 |
| Yes | 57 | 3 | 5.3 | 54 | 94.7 |  |
| No | 15214 | 1170 | 7.7 | 14044 | 92.3 |  |
| Mental and behavioral disorder, ICD: F00-F99 |  |  |  |  |  | 0.102 |
| Yes | 55 | 1 | 1.8 | 54 | 98.2 |  |
| No | 15216 | 1172 | 7.7 | 14044 | 92.3 |  |
| Nervous system diseases, ICD: G00-G99 |  |  |  |  |  | 0.591 |
| Yes | 468 | 39 | 8.3 | 429 | 91.7 |  |
| No | 14803 | 1134 | 7.7 | 13669 | 92.3 |  |
| Eye/ ear diseases, ICD: H00-H95 |  |  |  |  |  | <0.001 |
| Yes | 2831 | 80 | 2.8 | 2751 | 97.2 |  |
| No | 12440 | 1093 | 8.8 | 11347 | 91.2 |  |
| Circulatory system diseases, ICD: I00-I99 |  |  |  |  |  | 0.002 |
| Yes | 144 | 1 | 0.7 | 143 | 99.3 |  |
| No | 15127 | 1172 | 7.7 | 13955 | 92.3 |  |
| Respiratory system diseases, ICD: J00-J99 |  |  |  |  |  | <0.001 |
| Yes | 6000 | 177 | 3.0 | 5823 | 97.0 |  |
| No | 9271 | 996 | 10.7 | 8275 | 89.3 |  |
| Gastrointestinal tract diseases, ICD: K00-K93 |  |  |  |  |  | 0.001 |
| Yes | 576 | 24 | 4.2 | 552 | 95.8 |  |
| No | 14695 | 1149 | 7.8 | 13546 | 92.2 |  |
| Skin and subcutaneous tissue diseases, ICD: L00-L99 |  |  |  |  |  | 0.280 |
| Yes | 165 | 9 | 5.5 | 156 | 94.5 |  |
| No | 15106 | 1164 | 7.7 | 13942 | 92.3 |  |
| Musculoskeletal system/connective tissue diseases, ICD: M00-M99 |  |  |  |  |  | 0.001 |
| Yes | 211 | 3 | 1.4 | 208 | 98.6 |  |
| No | 15060 | 1170 | 7.8 | 13890 | 92.2 |  |
| Genitourinary system diseases, ICD: N00-N99 |  |  |  |  |  | 0.617 |
| Yes | 3 | 0 | 0 | 3 | 100 |  |
| No | 15268 | 1173 | 7.7 | 14095 | 92.3 |  |
| Congenital malformations and chromosomal abnormalities, ICD: Q00-Q99 |  |  |  |  |  | 0.031 |
| Yes | 140 | 4 | 2.9 | 136 | 97.1 |  |
| No | 15131 | 1169 | 7.7 | 13962 | 92.3 |  |
| Symptoms, signs, abnormal findings, ill-defined causes, not otherwise classified, ICD: R00-R99 |  |  |  |  |  | 0.105 |
| Yes | 849 | 53 | 6.3 | 796 | 93.7 |  |
| No | 14422 | 1120 | 7.8 | 13302 | 92.2 |  |
| Injury, poisoning and certain other consequences of external causes, ICD: S00-T98 |  |  |  |  |  | <0.001 |
| Yes | 808 | 32 | 4.0 | 776 | 96.0 |  |
| No | 14463 | 1141 | 7.9 | 13322 | 92.1 |  |
| Factors influencing good health and other utilization of the health care system, ICD: Z00-Z99 |  |  |  |  |  | 0.250 |
| Yes | 106 | 5 | 4.7 | 101 | 95.3 |  |
| No | 15165 | 1168 | 7.7 | 13997 | 92.3 |  |
| **PCCL** |  |  |  |  |  | <0.001 |
| High (0-1) | 1391 | 259 | 18.6 | 1132 | 81.4 |  |
| Low (2-4) | 11526 | 663 | 5.8 | 10863 | 94.2 |  |
| **DRG-Partition** |  |  |  |  |  | <0.001 |
| Surgical | 9554 | 623 | 6.5 | 8931 | 93.5 |  |
| Medical | 5632 | 542 | 9.6 | 5090 | 90.4 |  |
| **Comorbidity** |  |  |  |  |  | <0.001 |
| High (≥ 4 SD) | 7207 | 800 | 11.1 | 6407 | 88.9 |  |
| Low (< 4 SD) | 8064 | 373 | 4.7 | 7691 | 95.3 |  |
| **Number of inpatients** |  |  |  |  |  | <0.001 |
| High volume | 10518 | 939 | 8.9 | 9579 | 91.1 |  |
| Low volume | 4753 | 234 | 4.9 | 4519 | 95.1 |  |
|  | **Mean±SD** | **Mean±SD** | | **Mean±SD** | |  |
| Age, years | 47.99±24.22 | 58.84±17.08 | | 46.18±24.75 | | <0.001 |
| Treatment duration, days | 4.42±4.08 | 5.68±5.62 | | 4.31±3.90 | | <0.001 |
| Secondary diagnoses, n | 3.72±3.87 | 5.31±4.49 | | 3.59±3.77 | | <0.001 |

ICD = International Classification of Diseases; PCCL = Patient Clinical Complexity; SD = Secondary diagnoses
