# Supplementary material for: 30-day unplanned readmission rate in otolaryngology patients: A population-based study in Thuringia, Germany
Source: PLoS One. 2019 Oct 17;14(10):e0224146. doi: 10.1371/journal.pone.0224146 (PMC6797198; doi:10.1371/journal.pone.0224146)
Supplement: S3 Table — (DOCX) [file pone.0224146.s003.docx]

**S3 Table**

| **S3 Table. Multivariate analysis of risk factors for 30-day readmission** | | | |
| --- | --- | --- | --- |
| **Parameter** | **OR** | **95% CI** | **p** |
| Age, years | 0.994 | 0.989-0.998 | 0.006 |
| Duration primary treatment, days | 1.003 | 0.987-1.019 | 0.709 |
| Secondary diagnoses, n | 1.025 | 1.006-1.043 | 0.009 |
| Gender |  |  | 0.034 |
| Female | 1 |  |  |
| Male | 1.196 | 1.013-1.414 |  |
| **PCCL** |  |  | <0.001 |
| Low (0-1) | 1 |  |  |
| High(2-4) | 2.047 | 1.652-2.535 |  |
| **DRG-Partition** |  |  | <0.001 |
| Surgical | 1 |  |  |
| Medical | 1.394 | 1.176-1.653 |  |
| **Number of inpatients** |  |  | <0.001 |
| Low volume | 1 |  |  |
| High volume | 1.426 | 1.203-1.692 |  |
| **Localization of the primary disease** | | | |
| Pharynx/ Cavity of mouth |  |  | 0.038 |
| No | 1 |  |  |
| Yes | 1.326 | 1.016-1.730 |  |
| Larynx |  |  | 0.019 |
| No | 1 |  |  |
| Yes | 1.488 | 1.057-2.070 |  |
| Neck |  |  | 0.032 |
| Yes | 1 |  |  |
| No | 1.527 | 1.036-2.250 |  |
| Ear |  |  | <0.001 |
| No | 1 |  |  |
| Yes | 2.160 | 1.403-3.333 |  |
| Paranasal sinus |  |  | 0.438 |
| No | 1 |  |  |
| Yes | 1.183 | 0.774-1.808 |  |
| Face/ Skin |  |  | 0.002 |
| Yes | 1 |  |  |
| No | 1.825 | 1.245-2.673 |  |
| Other localization |  |  | 0.731 |
| Yes | 1 |  |  |
| No | 1.076 | 0.707-1.638 |  |
| **ICD-code** |  |  |  |
| Malignant diseases, ICD: C00-C97 |  |  | <0.001 |
| No | 1 |  |  |
| Yes | 5.556 | 7.143- 4.348 |  |
| Certain infectious and parasitic diseases, ICD: A00-B99 |  |  | 0.007 |
| Yes | 1 |  |  |
| No | 4.075 | 1.478-11.236 |  |
| Eye/ ear diseases, ICD: H00-H95 |  |  | <0.001 |
| Yes | 1 |  |  |
| No | 5.037 | 3.198-7.933 |  |
| Respiratory system diseases, ICD: J00-J99 |  |  | <0.001 |
| Yes | 1 |  |  |
| No | 2.412 | 1.745-3.333 |  |
| Gastrointestinal tract diseases, ICD: K00-K93 |  |  | 0.024 |
| Yes | 1 |  |  |
| No | 1.715 | 1.074-2.736 |  |
| Musculoskeletal system/connective tissue diseases, ICD: M00-M99 |  |  | 0.045 |
| Yes | 1 |  |  |
| No | 3.453 | 1.030-11.583 |  |
| Congenital malformations and chromosomal abnormalities, ICD: Q00-Q99 |  |  | 0.395 |
| Yes | 1 |  |  |
| No | 1.566 | 0.557-4.401 |  |
| Injury, poisoning and certain other consequences of external causes, ICD: S00-T98 |  |  | 0.042 |
| Yes | 1 |  |  |
| No | 1.620 | 1.019-2.577 |  |

OR = Odds ratio, CI = confidence interval; ICD = International Classification of Diseases; PCCL = Patient Clinical Complexity
