# Supplementary figures and images for: 30-day unplanned readmission rate in otolaryngology patients: A population-based study in Thuringia, Germany
Source: PLoS One. 2019 Oct 17;14(10):e0224146. doi: 10.1371/journal.pone.0224146 (PMC6797198; doi:10.1371/journal.pone.0224146)

**S1 Figure**


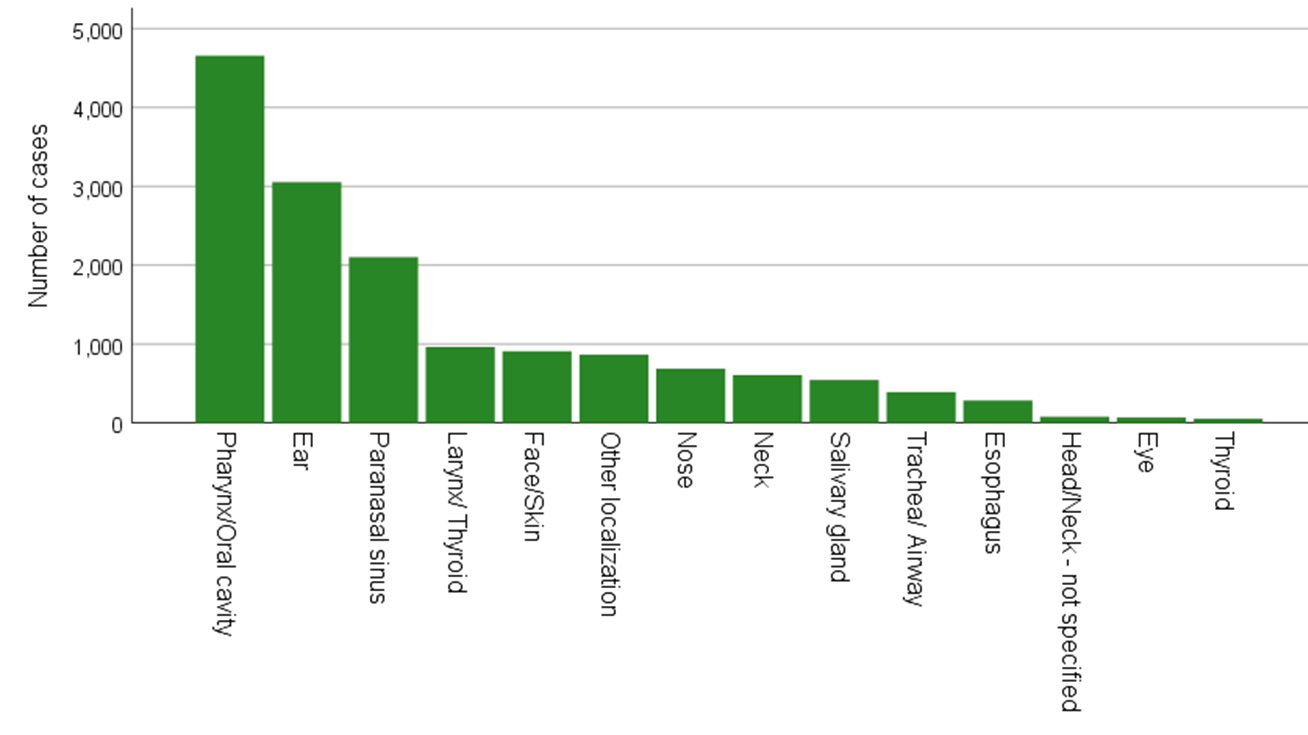

Supplement: S1 Fig — (DOCX) [file pone.0224146.s004.docx]

**S2 Figure**


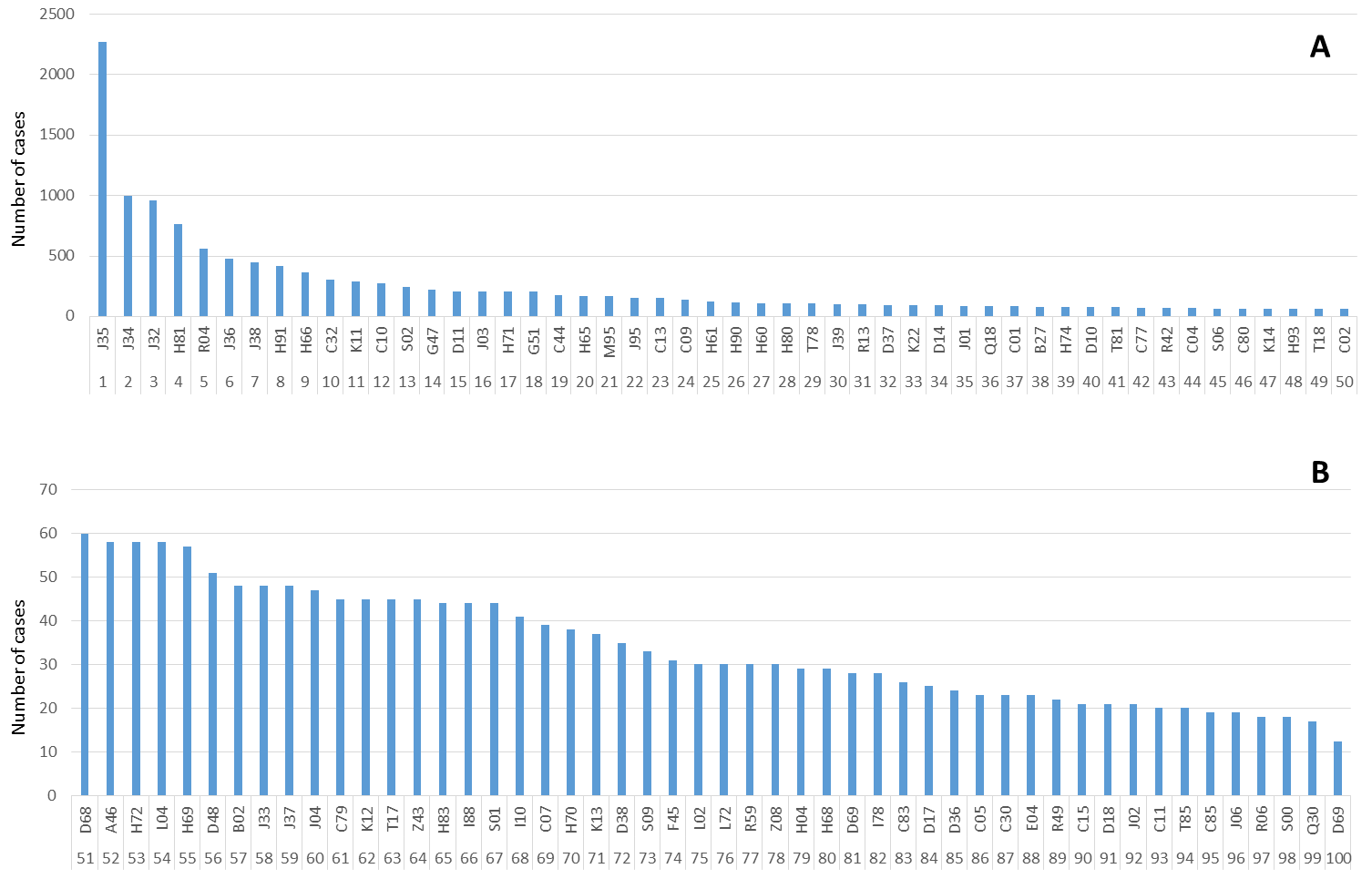

Supplement: S2 Fig — A: First 50 most frequent ICD codes. B: Second 50 most frequent ICD codes. (DOCX) [file pone.0224146.s005.docx]
