# Supplementary material for: 30-day unplanned readmission rate in otolaryngology patients: A population-based study in Thuringia, Germany
Source: PLoS One. 2019 Oct 17;14(10):e0224146. doi: 10.1371/journal.pone.0224146 (PMC6797198; doi:10.1371/journal.pone.0224146)
Supplement: Attachment — Submitted filename: Second rebuttal letter v4.docx [file pone.0224146.s006.docx]

**Rebuttal letter**

PONE-D-19-21224
30-day unplanned readmission rate in otolaryngology patients: a population-based study in Thuringia, Germany

Thank you very much for the second round of detailed reviews. Herewith, we would like to respond to all queries.

**1. Editorial comments**

1.1. Please give a concrete example of "planned re-admission".

Answer 1.1. We added some sentences for definition in the Methods on page 5: “Planned readmissions were defined as readmissions that were planned and agreed during the index admission. For instance, a patient with head and neck cancer had his index admission for staging and panendoscopy. Typically, if surgery was planned for definitive treatment, the day of readmission already was scheduled before demission. All other patients without planned readmission were defined as unplanned readmissions, for instance when a patient was readmitted for a complication after surgery.”.

1.2. Please double check the tables to ensure that no labels are missing. Some rows/columns are blank.

Answer 1.2. We checked all tables and supplement tables. There is no complete blank row or columns. The blank fields in many rows/columns are correct.

1.3. Please discuss risk factors for readmission in more detail.

Answer 1.3. See also answer 1.4. Including the paper of Dziegielewski et al 2016 already helped to discuss the risk factors in more detail. Furthermore, we added a paragraph in the Discussion on page 16/17 and discussed the most frequent reasons for unplanned readmission.

1.4. Please address how head and neck surgery patients may have affected the results. There is a paper (Dziegielewski et al 2016) that discuss this group of patients in detail. It would be worthwhile comparing results.

Answer 1.4. Thank you for this hint. The article of Dziegielewski et al. 2016 is now the new reference No. 17. The Discussion contains now a part on page 16 where we discuss this study in relation to our results. And we address this study in the Discussion on page 17 where we talk about the limitations of our study.

**2. Reviewer #1**

2.1. Overall the authors have improved the manuscript. I am still confused as to the concept of a "planned readmission" before discharge from the hospital. In what clinical scenarios would this happen? For a planned reconstruction surgery? For a second stage procedure? Can the authors provide an example of "planned readmission"? Otherwise other concerns have been addressed.

Answer 2.1. See also Answer 1.1. We added some sentences for the definition of planned and unplanned surgery on page 5 in the Methods.

**3. Reviewer #2**

3.1. The authors have adequately addressed each of the suggested revisions. My only remaining suggestion is to add x-axis labels to Figure 2 (e.g., "Number of cases" as in Figure 1).

Answer 3.1. Done. The x-axis in Figure 2 has now also a labeling: “Number of cases”.

**4. Reviewer #3**

4.1. Please correct all typographical errors (e.g., punctuation, capitalizatio of common nouns, etc). In the conclusions section, the authors may also want to state how the findings can be used to improve hospital discharge policies

Answer 4.1. The text was checked for typographical errors again. Concerning the improvement of the hospital discharge policies, the Conclusions contain three sentences: “Patients with the mentioned risk factors (and caregivers) should receive specific perioperative education programs addressing the related reasons that might lead to readmission. Prophylactic measures like prophylactic treatment if appropriate or a higher frequency of follow-up visits in the hand of one responsible physician might help to reduce the risk of unplanned 30-day readmission.”.

Orlando Guntinas-Lichius

for all authors

Jena, 22-September-2019
